# Sealing Behavior in Transcatheter Bicuspid and Tricuspid Aortic Valves Replacement Through Patient-Specific Computational Modeling

**DOI:** 10.3389/fcvm.2021.732784

**Published:** 2021-10-11

**Authors:** Xianbao Liu, Jiaqi Fan, Peter Mortier, Yuxin He, Qifeng Zhu, Yuchao Guo, Xinping Lin, Huajun Li, Jubo Jiang, Giorgia Rocatello, Vanda Oliveira, Tim Dezutter, Lars Sondergaard, Jian'an Wang

**Affiliations:** ^1^Department of Cardiology, Second Affiliated Hospital, Zhejiang University School of Medicine, Hangzhou, China; ^2^FEops, Ghent, Belgium; ^3^Department of Cardiology, Rigshospitalet, Copenhagen, Denmark

**Keywords:** transcatheter aortic valve replacement, bicuspid aortic valve, patient-specific computational modeling, sealing behavior, paravalvular leakage

## Abstract

**Background:** Patient-specific computer simulation of transcatheter aortic valve replacement (TAVR) can provide unique insights in device-patient interaction.

**Aims:** This study was to compare transcatheter aortic valve sealing behavior in patients with bicuspid aortic valves (BAV) and tricuspid aortic valves (TAV) through patient-specific computational modeling.

**Methods:** Patient-specific computer simulation was retrospectively performed with FEops HEARTguide for TAVR patients. Simulation output was compared with postprocedural computed tomography and echocardiography to validate the accuracy. Skirt malapposition was defined by a distance larger than 1 mm based on the predicted device-patient interaction by quantifying the distance between the transcatheter heart valve (THV) skirt and the surrounding anatomical regions.

**Results:** In total, 43 patients were included in the study. Predicted and observed THV frame deformation showed good correlation (*R*^2^ ≥ 0.90) for all analyzed measurements (maximum diameter, minimum diameter, area, and perimeter). The amount of predicted THV skirt malapposition was strongly linked with the echocardiographic grading of paravalvular leakage (PVL). More THV skirt malapposition was observed for BAV cases when compared to TAV cases (22.7 vs. 15.5%, *p* < 0.05). A detailed analysis of skirt malapposition showed a higher degree of malapposition in the interleaflet triangles section for BAV cases as compared to TAV patients (11.1 vs. 5.8%, *p* < 0.05).

**Conclusions:** Patient-specific computer simulation of TAVR can accurately predict the behavior of the Venus A-valve. BAV patients are associated with more malapposition of the THV skirt as compared to TAV patients, and this is mainly driven by more malapposition in the interleaflet triangle region.

## Introduction

Transcatheter aortic valve replacement (TAVR) in patients with a bicuspid aortic valve (BAV) is becoming increasingly important due to expanding indications which include younger patients, as well as to global adoption. In many countries, TAVR has become the standard of care for high-risk patients, and is now expanding into younger, lower-risk patients, resulting in an increased amount of patients with BAV stenosis (1–4). On the other hand, the Chinese TAVR market is still relatively small but growing rapidly, and the prevalence of BAV cases in China is notably higher than in other countries (5). Several clinical studies have demonstrated the safety and efficacy of TAVR in BAV patients (3, 4), but there are still several challenges when treating BAV stenosis and patients should be carefully selected. Therefore, efforts to increase our knowledge of how TAVR devices interact with BAVs remain important.

The interaction of transcatheter heart valves with the aortic root is likely to be different between BAV and tricuspid aortic valve (TAV) patients. While device sizing for TAV cases is mainly based on the dimensions of the aortic annulus, an assessment of the supra-annular structure seems mandatory for BAV cases as this can be the primary location where the THV interacts with the aortic root (6, 7). This, however, depends on several anatomical factors such as BAV type, calcium burden, raphe length, and the ratio of the intercommissural diameter to the mean annular diameter (7).

Patient-specific computational modeling of TAVR with FEops HEARTguide (FEops, Ghent, Belgium) based on pre-procedural computed tomography (CT) has emerged as a promising technology capable of accurately predicting device-anatomy interaction, as well as paravalvular leakage and the risk on TAVR-induced conduction abnormalities for both TAV and BAV patients (8–12). Validation data are mainly available for the Medtronic self-expanding and the Boston Scientific mechanically expandable THVs. These three-dimensional computer models provide detailed insights that cannot be obtained through post-procedural imaging, and may also help to better understand the sealing behavior in BAV and TAV patients.

In this study, we aimed to validate a patient-specific computer simulation of TAVR in Chinese patients treated with the self-expandable Venus A-valve and use the validated computational model to explore potential differences in the sealing behavior between BAV and TAV patients.

## Methods

A retrospective single-center study was performed on patients who underwent transcatheter aortic valve replacement using a Venus A-valve (Venus Medtech). Both pre- and post-procedural CT imaging was available for all patients. All dual source computed tomography (DSCT) examinations were performed with the second generation dual-source CT (SOMATOM Definition Flash, Siemens Medical Solutions, Germany). The scan area was craniocaudal from the subclavian artery to the iliofemoral branches. Prospective ECG gating with a pitch of 2.4 was performed. Around 60–80 ml of iodine-containing contrast agent (Omnipaque 370 mg I/ml, GE Healthcare, Shanghai, China) was injected with a dual-head power injector (Mallinckrodt, American) at a flow rate of 4 ml/s followed by 60 ml of 0.9% saline solution at the same flow rate. A bolus tracking method was used in the descending aorta with a pre-set threshold of 180 Hounsfield units (HU) to achieve optimal synchronization. The tube voltage was 100 kV, with a reference tube current-time product of 280 mAs and a collimation of 38.4 mm (2^*^32^*^0.6 mm^3^) with double sampling by a z-axis flying focal spot. All procedures were performed as reported in previous studies (13, 14). The study was approved by the medical ethics committee of Second Affiliated Hospital of Zhejiang University and carried out according to the principles of the Declaration of Helsinki. All patients provided written informed consent for TAVR and the use of anonymous clinical, procedural, and follow-up data for research.

### Virtual Device Modeling

Accurate finite element models of the frames of all Venus A-valve sizes (23, 26, 29, and 32 mm) were generated based on CAD (Computer Aided Design) data provided by the device manufacturer. A virtual radial force test was performed to validate the virtual device models using the finite element analysis (FEA) software Abaqus (Abaqus v6.12, Dassault Systèmes, Paris, France). For this test, the device was crimped to a smaller diameter (loading) and then released (unloading) while the radial force in the crimper was measured. The model radial force was then compared with the experimental radial force data during unloading and within the relevant deployment range for each valve size. Model parameters were calibrated until excellent agreement was obtained. A mesh density analysis was performed on the device radial force to determine the optimal number of elements, which is around 4,000 elements for the different device sizes.

### Patient-Specific Computational Modeling

Three-dimensional patient-specific geometries of the native aortic root (including the calcified native leaflets) were reconstructed from pre-operative contrast-enhanced CT scans, using the image segmentation software Mimics (Mimics v21.0, Materialise, Leuven, Belgium). The aortic wall and the calcified leaflets are modeled using ~15,000 and 7,000 elements, respectively. Different material behavior was automatically assigned to the different tissue regions. Linear elastic models rather than more realistic but also more complex hyperelastic anisotropic models were adopted to describe the aortic root tissues. These simplified material models facilitated deriving the material parameters in a previous study with an iterative process of back-calculations using pre- and postoperative MSCT of 39 patients (8). For the aortic wall, an elastic modulus of 2 MPa and a uniform thickness of 2 mm was used, while for the aortic leaflets, an elastic modulus of 0.6 MPa and uniform thickness of 1.5 mm was adopted. Calcifications were modeled using a stiffer elastic material with perfect plasticity (E = 4 MPa, yield stress = 0.6 MPa).

Venus A-valve models were then virtually deployed in these geometries using Abaqus (v6.12, Dassault Systemes, Simulia Corp, Johnson, RI). These simulations allow us to assess the device, native leaflet, and aortic wall deformation as previously described (8, 10, 11). The simulation strategy consists of a number of steps. The device is first crimped to a small diameter using a cylindrical surface. Then it is positioned nearly co-axially within the aortic root, and deployed by retracting a catheter. The default general contact with finite sliding between all the surfaces was used, assuming a coefficient of friction of 0.7 between the valve frame and the aortic model.

**Figure 1:**
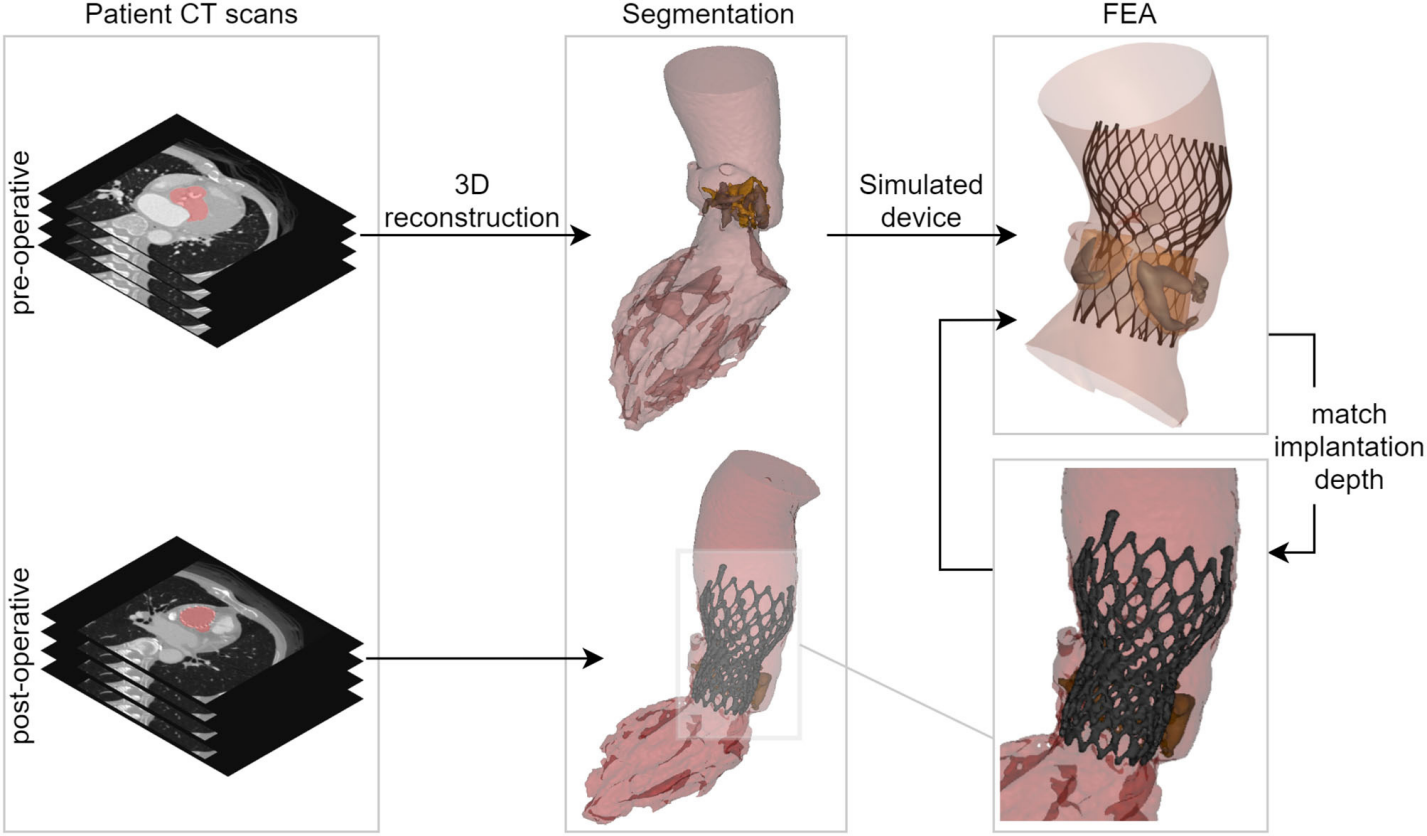
Overview showing the main steps of the virtual insertion of a Venus A-valve in patient-specific geometry derived from pre-operative CT, while aiming for a virtual implantation depth identical to the actual one. CT, computed tomography; 3D, three-dimensional; FEA, finite element analysis.

For each simulated implantation, the valve size selection and the depth of implantation were aligned with the clinical procedure. The simulated depth of implantation was iteratively adjusted to match the actual depth of implantation derived from the post-operative geometry, which was reconstructed from post-operative CT images using Mimics. This was done by overlaying the simulation results with the post-operative geometry using a manual geometrical registration method. In case the simulated device position differed from the observed position (post-op MSCT), an additional iteration was performed until a satisfactory match in terms of implantation depth was obtained. An overview of these different reconstruction and modeling steps is summarized in Figure 1.

### Frame Deformation Comparison

For each patient, predicted frame deformation was both qualitatively and quantitatively compared to the post-operative device deformation (CT). A visual inspection was performed by overlaying the predicted and post-operative devices, and their dimensions (minimum and maximum diameter, perimeter, and area) were quantified at four relevant device levels (Supplementary Figure 1): commissures, central coaptation, nadir, and ventricular end (8).

### Sealing Analysis

The regions of skirt apposition and malapposition were determined for all patients using the predicted device and aortic root deformation. Apposition was considered when the deformed device skirt was in contact with the anatomy, while malapposition was considered when the opposite was verified. The areas corresponding to the apposed and malapposed skirt were quantified in four different regions of the aortic root anatomy: left ventricular outflow tract (LVOT), leaflets, interleaflet triangles, and ascending aorta.

**Figure 2:**
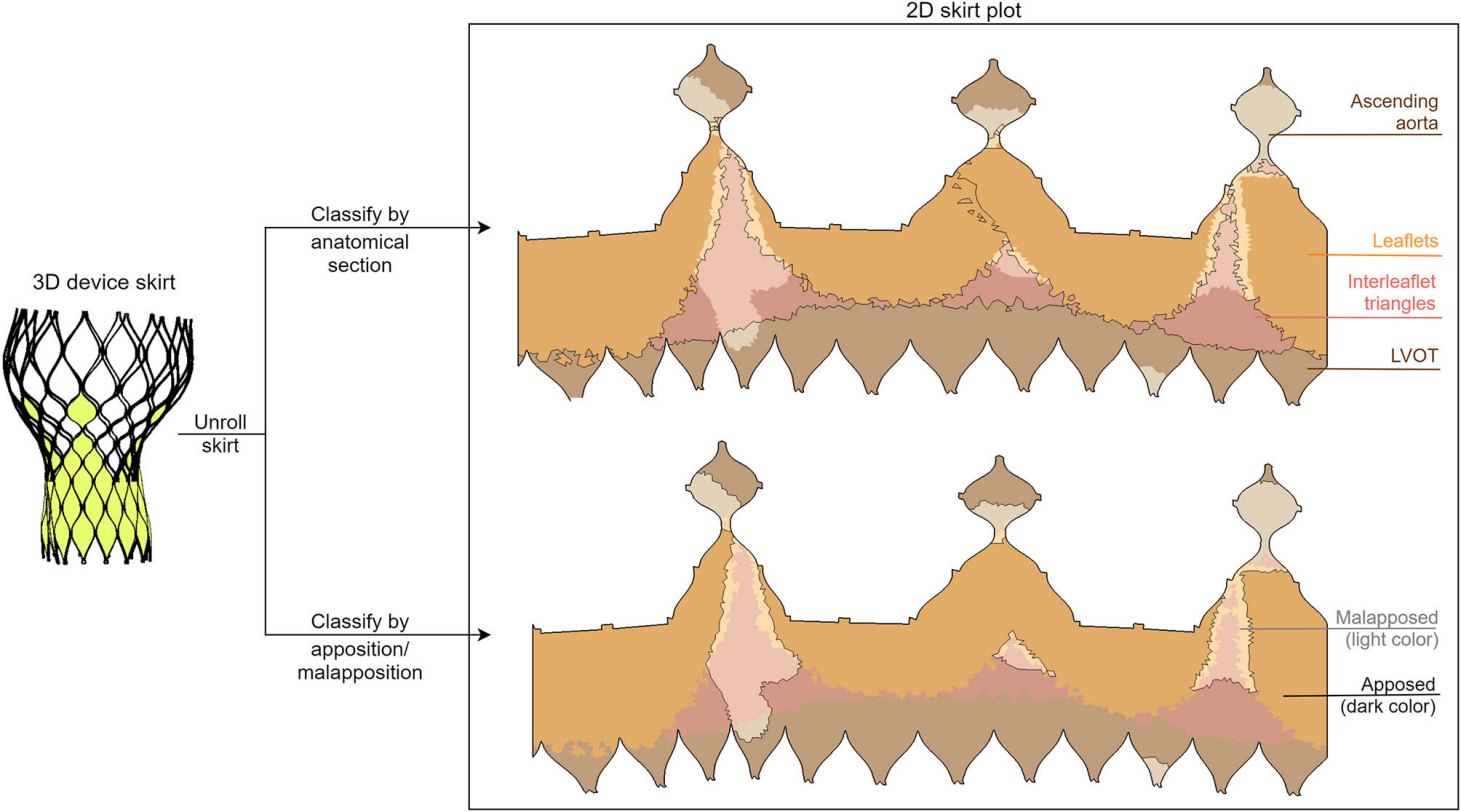
Illustration of the division of the two-dimensional deformed device skirt into anatomical (top) and apposition/malapposition (bottom) sections. This division is evidenced both with a border line and with a color palette, as depicted in the legend.

In order to obtain these regions, the deformation anatomy (after simulated device deployment) was firstly divided into the anatomical sections mentioned above. Then, each element of the simulated skirt was attributed to one of these anatomical regions and the distance between the skirt and the anatomy was calculated. This was done by searching the anatomy element in the normal direction to each skirt element. Apposition and malapposition were then attributed to each element based on the distance (apposed if the distance was smaller than 1 mm, malapposed otherwise). Finally, the skirt was projected in 2D and the apposed and malapposed areas were computed for each anatomical section. A visual overview of the separation of the skirt into sections (both anatomical and apposition) is shown in Figure 2. The obtained area values were grouped according to the aortic valve morphology: tricuspid (TAV), bicuspid (all types, BAV), bicuspid type 0 (BAV0), and BAV type 1 (BAV1) using the Sievers classification (15).

### PVL Comparison

Transthoracic Doppler echocardiography was used for the clinical PVL assessment. PVL was classified as none or trace, mild, or moderate based on the VARC-2 criteria. Observed PVL grades were compared to the predicted amount of skirt malapposition. The grades were also divided per valve morphology to detect possible patterns between the PVL severity and valve morphology.

### Statistical Analysis

Continuous variables are expressed as mean ± SD. Correlation between predicted and observed continuous variables was analyzed using the coefficient of determination (*R*^2^). Comparisons within the sealing analysis were carried out using the paired Student *t*-test or Mann–Whitney U-test depending on the variable distribution. Baseline characteristics and anatomic parameters were analyzed to explore the association with malapposition in interleaflet triangles. Only variables yielding a *p*-value < 0.1 were included in the stepwise multivariate linear regression analysis. Statistical significance was defined as a two-tailed *p* < 0.05. Statistical analysis was performed with SciPy Stats, a Python module for probability functions and statistical distributions.

## Results

A total of 43 patients were included in the study. There was no significant difference in age between BAV and TAV patients (BAV: 76.4 ± 7.1 years old vs. TAV: 79.4 ± 6.2 years old, *p* = 0.164) or other baseline characteristics (Table 1). Of these 43 patients, 26 patients were BAV patients of which 11 patients were type 0 and 15 were type 1. For the BAV patients, the sizing index (ratio of device size to perimeter-derived diameter) was lower when compared with TAV patients (BAV: 1.04 ± 0.09 vs. TAV: 1.11 ± 0.07, *p* = 0.018).

**Table 1:** Patient characteristics.

	**BAV type 0**	**BAV type 1**	**BAV**	**TAV**	
	***n*** **= 11**	***n*** **= 15**	***n*** **= 26**	***n*** **= 17**	***p*** **-value**
Age (yrs)	77.8 ± 5.6	75.4 ± 8.1	76.4 ± 7.1	79.4 ± 6.2	0.164
Male	5 (45.5)	12 (80.0)	17 (65.4)	9 (52.9)	0.528
Height (cm)	159.1 ± 6.5	165.7 ± 6.5	162.9 ± 7.2	162.2 ± 8.8	0.780
Weight (kg)	59.0 ± 8.0	64.9 ± 9.4	62.4 ± 9.1	60.4 ± 11.8	0.544
Body Mass Index (kg/m^2^)	23.31 ± 3.01	23.59 ± 2.94	23.47 ± 2.91	22.85 ± 3.59	0.542
STS	5.29 ± 3.12	5.15 ± 2.56	5.21 ± 2.75	8.33 ± 5.95	0.124^*^
**Echocardiography**					
Left ventricular ejection fraction (%)	54.9 ± 14.9	55.0 ± 12.7	55.0 ± 13.3	54.8 ± 17.3	0.593^*^
Aortic valve area (cm^2^)	0.53 ± 0.21	0.66 ± 0.15	0.60 ± 0.19	0.62 ± 0.21	0.726
Mean gradient (mmHg)	59.4 ± 19.4	56.5 ± 15.2	57.7 ± 16.8	52.9 ± 11.5	0.478^*^
Max velocity (m/s)	4.98 ± 0.90	4.67 ± 0.94	4.80 ± 0.92	4.70 ± 0.42	0.526^*^
**Multi-slice computed tomography**					
Max annulus diameter (mm)	27.4 ± 3.5	29.6 ± 3.0	28.7 ± 3.4	27.4 ± 3.2	0.240
Min annulus diameter (mm)	21.7 ± 3.5	22.7 ± 3.4	22.3 ± 3.4	21.1 ± 2.3	0.209
Mean annulus diameter (mm)	24.5 ± 3.4	26.2 ± 3.1	25.5 ± 3.3	24.3 ± 2.7	0.212
Perimeter derived diameter (mm)	24.7 ± 3.3	26.5 ± 3.1	25.8 ± 3.3	24.7 ± 3.0	0.312
Area derived diameter (mm)	24.3 ± 3.3	26.0 ± 3.1	25.3 ± 3.2	24.2 ± 2.9	0.285
Calcium volume (mm^3^)	1210.7 ± 778.0	1213.5 ± 676.6	1212.4 ± 701.6	781.8 ± 576.6	0.094
**Procedural characteristics**					
Implanted depth (mm)	6.2 ± 3.3	6.1 ± 4.2	6.1 ± 3.7	7.4 ± 3.1	0.236
Device size					0.586
23 mm	3 (27.3)	1 (6.7)	4 (15.4)	1 (5.9)	
26 mm	7 (63.6)	8 (53.3)	15 (57.7)	11 (64.7)	
29 mm	0 (0.0)	5 (33.3)	5 (19.2)	2 (11.8)	
32 mm	1 (9.1)	1 (6.7)	2 (7.7)	3 (17.6)	
Sizing index^§^	1.05 ± 0.11	1.03 ± 0.09	1.04 ± 0.09	1.11 ± 0.07	0.018
**Post-procedural outcomes**					
Mortality	0 (0.0)	0 (0.0)	0 (0.0)	0 (0.0)	–
Stroke	0 (0.0)	0 (0.0)	0 (0.0)	0 (0.0)	–
MI	0 (0.0)	0 (0.0)	0 (0.0)	0 (0.0)	–
PVL III/IV	3 (27.3)	2 (13.3)	5 (19.2)	1 (5.9)	0.376
Pacemaker implantation	1 (9.1)	1 (6.7)	2 (7.7)	2 (11.8)	1.000

§ *Sizing index, (device size)/(perimeter-based diameter)*.

* *Mann-Whitney U-test was used*.

*Data are presented as no. (%) and mean ± SD*.

*BAV, bicuspid aortic valve; MI, myocardial infarction; PVL, paravalvular leakage; TAV, tricuspid aortic valve*.

### Comparison of Observed and Predicted Parameters

**Figure 3:**
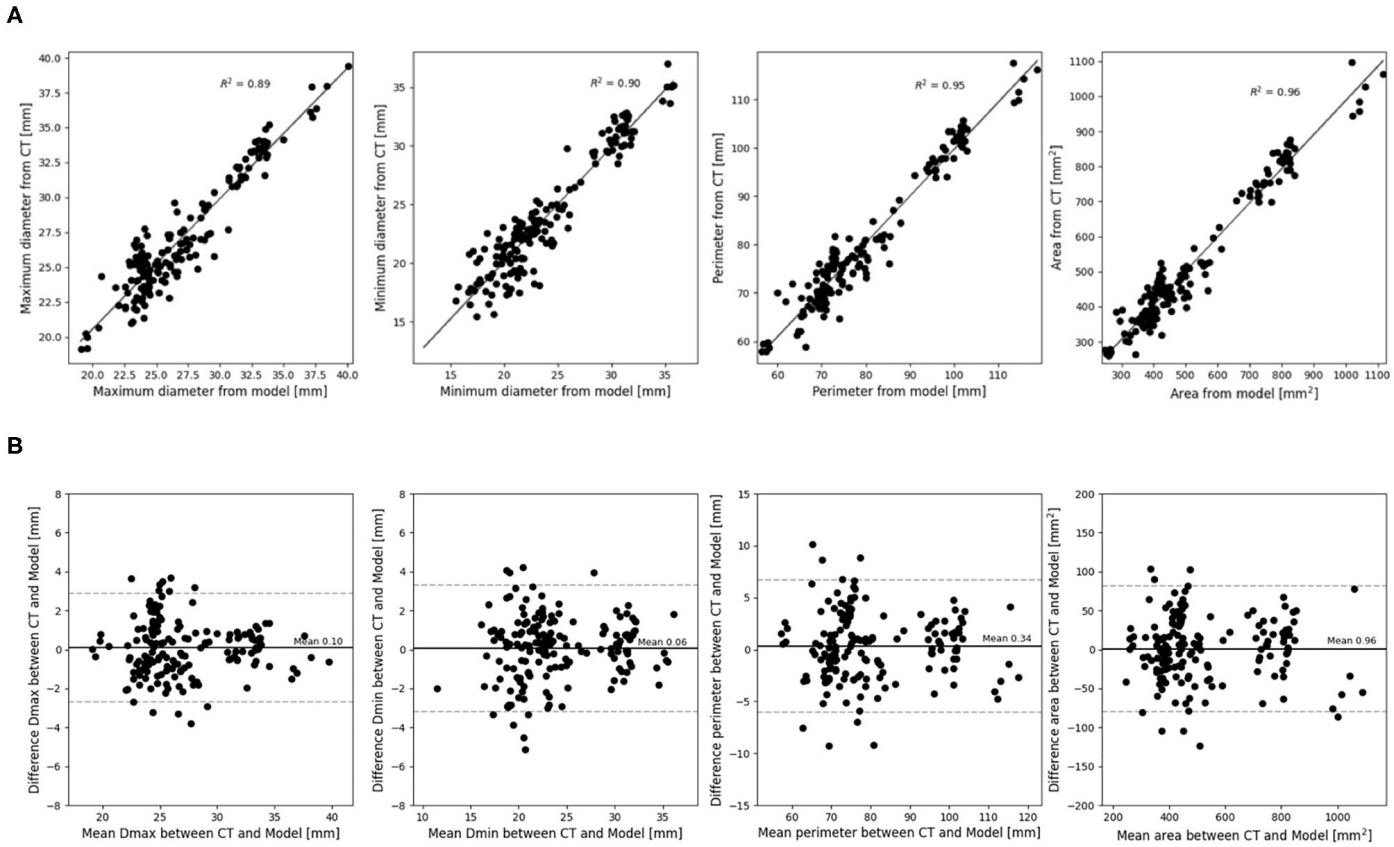
**(A)** Correlation and **(B)** difference plots for obtained device measurements vs. predicted device measurements for all device levels.

The mean differences and coefficients of determination between the measurements extracted from the post-operative and simulated device are summarized in Table 2. This is presented for each type of measurement for all levels of the device combined. A high coefficient of determination was obtained for all measurements (≥0.90). All dimensions were slightly underestimated by the model, but the mean differences are negligible. Correlation and difference plots for each type of measurement are presented in Figures 3A,B.

**Table 2:** Mean (±SD) difference between the measurements of the observed (post-operative) and simulated (model) devices and respective R-squared coefficient for the different levels of the devices.

**Measurement**	**Mean difference (Post-op - Model)**	***R* ^2^**
Dmax (mm)	0.10 ± 1.42	0.90
Dmin (mm)	0.06 ± 1.55	0.90
Perimeter (mm)	0.34 ± 3.25	0.95
Area (mm^2^)	0.96 ± 41.14	0.96

**Figure 4:**
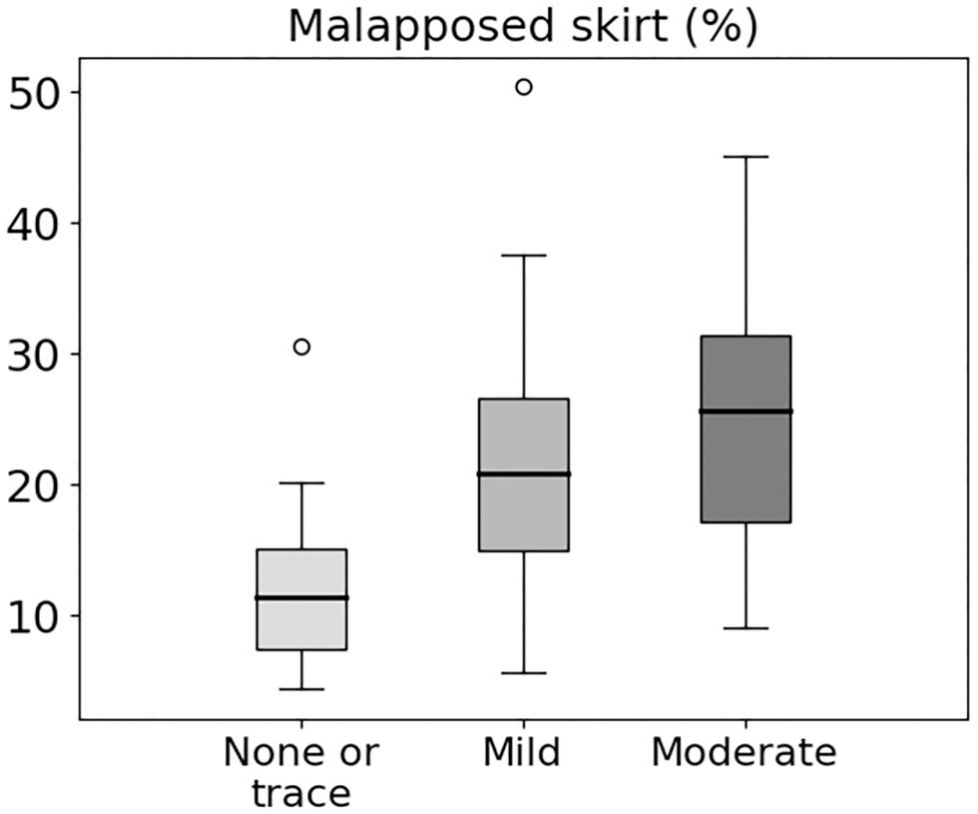
Box and whiskers diagram showing predicted skirt malapposition for patients with none or trace, mild, and moderate PVL. Extreme values are presented as small circles (o).

Echocardiography showed none or trace post-operative PVL in 13 patients, mild PVL in 24, and moderate PVL in 6. Figure 4 shows a comparison of predicted skirt malapposition for patients with none to trace, mild, and moderate PVL. The amount of skirt malapposition is higher for patients with a higher degree of clinically assessed PVL (Supplementary Table 1).

**Figure 5:**
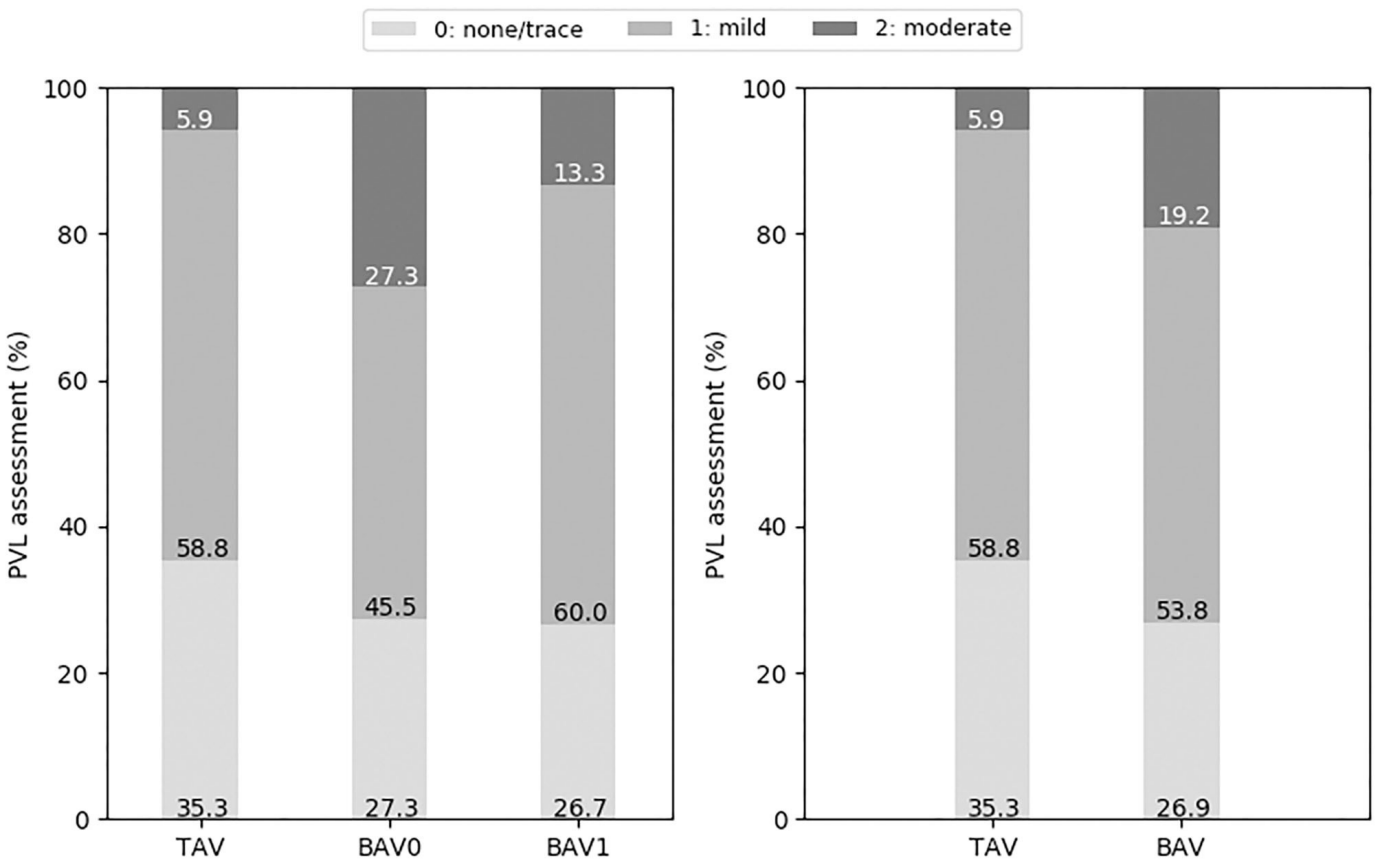
Post-operative PVL grading (none or trace, mild, and moderate) for the different types of valve morphologies: TAV, BAV, BAV0, and BAV1.

A comparison of post-operative PVL assessment for patients with different valve morphologies is shown in Figure 5. Moderate PVL was more frequent for BAV cases (19.2 vs. TAV 5.9%), with BAV0 having the highest incidence of moderate PVL (27.3% for BAV0 vs. 13.3% for BAV1, respectively).

### Comparison of Sealing Behavior

**Figure 6:**
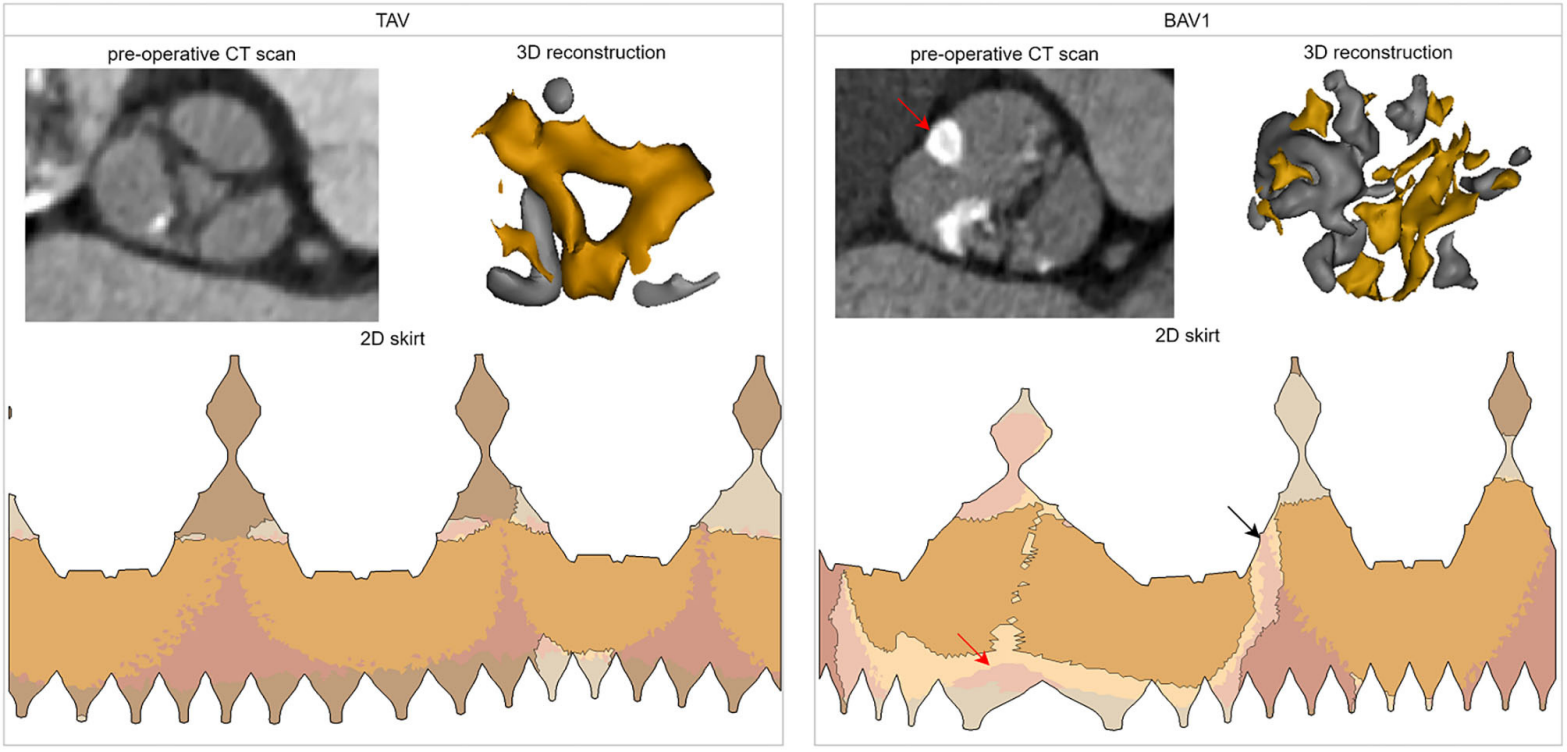
Analysis of two cases of the cohort with different valve morphologies: TAV (total skirt malapposition of 4.4%, PVL grade 0) and BAV1 (total skirt malapposition of 20.9%, PVL grade 1). For each case, the following is shown: cross-section at the aortic annular plane in the pre-operative CT, three-dimensional reconstruction of the valve (and its calcifications), and distribution of the apposition/malapposition sections of the two-dimensional deformed device skirt. For the BAV1 case, the red arrow indicates the raphe and the black arrow the largest PVL channel. CT, computed tomography; 3D, three-dimensional; 2D, two-dimensional.

Representative TAV (total skirt malapposition of 4.4%, no PVL) and BAV1 (total skirt malapposition of 20.9%, mild PVL) cases are depicted in Figure 6. A cross-section of the pre-operative CT scan at the aortic annular plane and a 3D reconstruction illustrate the morphology of the valves. For each valve, the 2D skirt is also shown with the apposition borders highlighted, evidencing the larger area of malapposition in the BAV1 case (relatively to TAV). For the BAV1 case, PVL channels are visible in the interleaflet triangles region.

**Figure 7:**
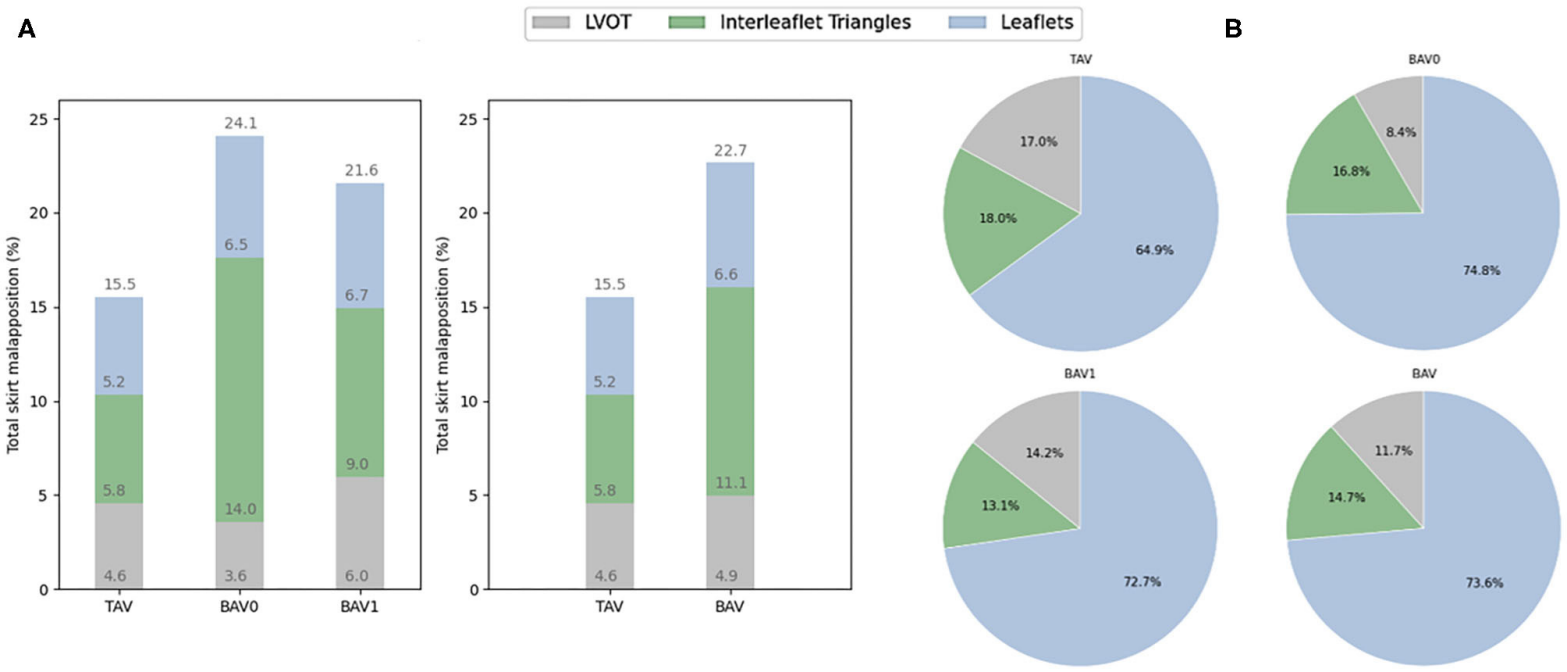
**(A)** Contribution of each anatomical section to the malapposition (mean, %) of the total skirt. **(B)** Contribution of each anatomical section to the apposition (mean, %) for the apposed section of the skirt. All these values are presented for the different types of valve morphologies: TAV, BAV, BAV0, and BAV1. TAV, tricuspid aortic valve; BAV, bicuspid aortic valve; BAV0, type 0 bicuspid aortic valve; BAV1, type 1 bicuspid aortic valve.

An overview of all sealing analysis data for each valve morphology is provided in Table 3. The mean percentage of total skirt malapposition obtained for each anatomical section (LVOT, interleaflet triangles and leaflets) relatively to the total skirt is illustrated as bar plots for the different valve morphologies in Figure 7A. In this analysis, the values obtained for the ascending aorta section were not considered to simplify the analysis. More malapposition was obtained for BAV cases when compared to TAV cases (22.7 vs. 15.5%, *p* < 0.05), and this is also true when comparing TAV cases to BAV type 0 and BAV type 1 cases separately. This seems mainly driven by a higher degree of malapposition in the interleaflet triangles section: 5.8 and 11.1% for TAV and BAV (*p* < 0.05), respectively.

**Table 3:** Overview of sealing analysis data for the different valve morphologies.

	**BAV type 0**	**BAV type 1**	**BAV**	**TAV**	
	***n*** **= 11**	***n*** **= 15**	***n*** **= 26**	***n*** **= 17**	***p*** **-value**
**LVOT**					
Apposed area (mm^2^)	72.4 ± 68.4	133.1 ± 190.4	107.4 ± 152.0	157.4 ± 135.7	0.106^*^
Malapposed area (mm^2^)	47.8 ± 87.0	81.6 ± 126.4	67.3 ± 110.7	52.0 ± 79.9	0.980^*^
**Interleaflet triangles**					
Apposed area (mm^2^)	146.4 ± 95.2	123.8 ± 75.5	133.4 ± 83.4	175.1 ± 73.4	0.100
Malapposed area (mm^2^)	146.1 ± 60.1	100.5 ± 45.7	119.8 ± 56.1	65.5 ± 36.4	0.001
**Leaflets**					
Apposed area (mm^2^)	590.6 ± 84.2	628.3 ± 191.8	612.4 ± 154.3	611.5 ± 193.6	0.987
Malapposed area (mm^2^)	69.1 ± 52.0	73.8 ± 30.6	71.8 ± 40.1	56.8 ± 30.1	0.195
**Malapposition in total skirt (%)**	24.1 ± 10.6	21.6 ± 10.5	22.7 ± 10.5	15.5 ± 9.8	0.030
Malapposition in LVOT (%)	3.6 ± 5.4	6.0 ± 8.2	4.9 ± 7.1	4.6 ± 7.1	0.960^*^
Malapposition in Interleaflet triangles (%)	14.0 ± 6.4	9.0 ± 4.0	11.1 ± 5.7	5.8 ± 2.8	0.001^*^
Malapposition in Leaflet (%)	6.5 ± 4.9	6.7 ± 3.1	6.6 ± 3.8	5.2 ± 2.9	0.200
**Apposition in total skirt (%)**	75.9 ± 10.6	78.4 ± 10.6	77.3 ± 10.5	84.5 ± 9.8	0.030
Apposition contribution LVOT (%)	8.4 ± 7.9	14.2 ± 19.8	11.7 ± 15.9	17.0 ± 16.0	0.124^*^
Apposition contribution Interleaflet triangles (%)	16.8 ± 8.7	13.1 ± 7.0	14.7 ± 7.9	18.0 ± 4.9	0.172^*^
Apposition contribution Leaflet (%)	74.8 ± 14.0	72.7 ± 20.0	73.6 ± 17.4	64.9 ± 17.6	0.120

* *Mann-Whitney U-test was used*.

*Data are presented as mean ± SD*.

*BAV, bicuspid aortic valve; LVOT, left ventricular outflow tract; TAV, tricuspid aortic valve*.

The percentage of apposed skirt obtained out of the apposed section of the total skirt is illustrated as pie charts for each anatomical section and valve morphology in Figure 7B. There is a trend for a higher contribution of the left ventricular outflow tract (LVOT) to the apposition in TAV (17.0%) as compared to BAV cases (11.7%), and this difference is most pronounced for BAV type 0 patients (8.4%). In contrast, the leaflets seem to contribute more to apposition in BAV (73.6%) than in TAV cases (64.8%), and this is also true when looking at BAV type 0 and type 1 separately. However, no statistical significance was observed for these comparisons.

The multivariate linear regression analysis identified BAV (*p* = 0.009) and the sizing index (*p* = 0.034) as two independent predictors of malapposition in interleaflet triangles. The results of univariate and multivariate linear regression for association with malapposition in interleaflet triangles are presented in Supplementary Table 2.

## Discussion

In this study, we evaluated a patient-specific computer model of TAVR in Chinese BAV and TAV patients with the self-expanding Venus A-valve using FEops HEARTguide (FEops, Ghent, Belgium). We compared the predicted THV frame deformation with postprocedural CT and found excellent correlation. Moreover, we observed a good agreement between predicted THV skirt malapposition and postoperative PVL based on echocardiography. Finally, we conducted a detailed sealing analysis and found that more malapposition was obtained in BAV patients when compared to TAV patients which was mainly driven by more malapposition at the location of the interleaflet triangles. Interestingly, the leaflets seem to be the main contributor to device sealing (apposition) not only in BAV but also in TAV cases.

### Validation of the Modeling

Patient-specific computer simulation of TAVR has been previously described and validated, not only in TAV patients but also in BAV cases (FEops, Ghent, Belgium) (8–12). These previous studies showed that computer simulation can accurately predict the THV frame deformation, severity of PVL, and potential occurrence of conduction abnormalities. However, these studies primarily focused on the Medtronic self-expanding and the Boston Scientific mechanically expandable THVs, and were all conducted by European hospitals. In this study, we employed the patient-specific computer simulation for the first time in a Chinese patient population with the self-expanding Venus A-valve. Despite the higher radial force of the Venus A-valve and the high calcium burden in this Chinese population, an excellent agreement between the predicted and observed dimensions of the valve frame was obtained (5, 16). Moreover, we compared predicted THV skirt malapposition and clinically assessed PVL, and found a good relationship.

These validated patient-specific computer simulations may help clinicians to better understand the risk of the procedure, and to optimize device sizing and positioning for each individual. This useful tool can also assist physicians recognizing patients who would benefit from TAVR and other patients for whom SAVR may be the preferred treatment. TAVR in mainland China is rapidly evolving, and the most widely used commercial THV is currently the Venus A-valve (17, 18). Therefore, the presented study may be an important step to bring this technology to Chinese physicians.

### Sealing Behavior in BAV and TAV Patients

TAVR in BAV patients has proven to be safe and effective, but patients need to be selected carefully and a widely accepted THV sizing strategy is still lacking. One key challenge of BAV disease is the increased anatomical heterogeneity as compared to TAV disease. In addition, there are important ethnic differences. In European populations, BAV type 1 with L-R coronary cusp fusion is most common, while in Asian populations, an unexpected high prevalence of type 0 was found (19, 20). As the deformed device skirt mainly interacts with three different regions of the aortic root anatomy, LVOT, leaflets, and interleaflet triangles, we performed a detailed analysis of the sealing behavior in these anatomical regions in BAV and TAV patients.

In the presented study, we found more malapposition in BAV patients when compared to TAV patients which was mainly driven by more malapposition in the interleaflet triangles. It should be emphasized that the pathologic landmark of BAV is always an absent or underdeveloped interleaflet triangle: a dysmorphic, underdeveloped interleaflet triangle is usually accompanied by a raphe, while the type 0 BAV is a valve with complete absence of one interleaflet triangle (21). Another factor is that TAVR in BAV might result in uneven bioprosthetic valve frame expansion after THV deployment, and the deformed device (skirt) may not touch the interleaflet triangle under the restricted stent-frame expansion (19, 22–24). This may explain the higher amount of malapposition that we observed in the interleaflet triangle region in type 0 and type 1 BAV cases compared with TAV.

Another finding is that the leaflets seem to be the main contributor to device sealing, not only in BAV but also in TAV cases. The importance of the interaction between the supra annular structure and THV has already been discussed in previous studies (7, 14, 25–27). Our present study based on patient-specific computer simulation further clarifies the crucial contribution of the leaflets to supra-annular sealing. Overall, these results confirm that an assessment of the supra-annular structure is important for the adequate planning of TAVR in BAV cases.

Moreover, in our present study, we found a trend for a higher contribution of the LVOT to the apposition in TAV as compared to BAV cases, and this difference was most pronounced for BAV type 0 patients. This result may be partially explained by the depth of implantation. As described in the baseline characteristics, BAV type 0 patients had a tendency of higher implantation than TAV. In addition, for BAV type 0 patients, the fish mouth-like shape of the valve may result in an under expansion of the THV frame in the annular and sub-annular (LVOT) region, and thus reduce the device-tissue interaction in this region.

### Malapposition and PVL

The presented sealing analysis based on computational modeling may reflect the risk of PVL after TAVR. As showed in Figure 4, a higher amount of malapposition seems related to the echo-based PVL grading. In addition, we observed a higher prevalence of moderate echocardiographic-identified PVL in the BAV group which might be explained by the observed difference in sealing behavior between BAV and TAV cases. Understanding the sealing behavior of TAVR in BAV and TAV patients could assist physicians to comprehensively assess the risk of PVL and evaluate the interaction between supra-annular structure and THV stent frame.

## Limitations

This study was a small and retrospective single-center study. Due to the low number of patients with more than moderate PVL, no formal statistical analysis was performed. More cases should be included to assess the sealing behavior on different leaflet fusion patterns. As a retrospective study, transthoracic echocardiography was used to assess the clinical PVL, and the location of PVL could not be evaluated due to the limitation of imaging quality.

## Conclusions

Patient-specific computer simulation of TAVR can be used in Chinese patients with the self-expanding Venus A-valve. Transcatheter aortic valve sealing behavior is different between BAV and TAV patients with more malapposition at the location of the interleaflet triangles section for BAV cases.

## Impact on Daily Practice

BAV patients are associated with more malapposition of the transcatheter heart valve (THV) skirt as compared to TAV patients, and this is mainly driven by more malapposition in the interleaflet triangle region. More cases and studies are needed to confirm the results and related malapposition of the THV skirt to clinical echo-based paravalvular leakage grading. Patient-specific computational modeling of TAVR based on pre-procedural CT might be performed in BAV patients. Transcatheter heart valve size and ideal implanted depth could be recommended to reduce the malapposition and potential paravalvular leakage.

## Data Availability Statement

The data analyzed in this study is subject to the following licenses/restrictions: dataset can only be accessed after getting permission. Requests to access these datasets should be directed to jqfan@zju.edu.cn.

## Ethics Statement

The studies involving human participants were reviewed and approved by the Medical Ethics Committee of Second Affiliated Hospital of Zhejiang University. The patients/participants provided their written informed consent to participate in this study. Written informed consent was obtained from the individual(s) for the publication of any potentially identifiable images or data included in this article.

## Author Contributions

JW, XLiu, and JF design the study. XLiu, JF, YH, QZ, YG, XLin, HL, and JJ conducted the study and performed the examinations. PM, GR, VO, TD, and JF performed the patient-specific computer simulation and statistical analysis. JF and PM performed the statistical analysis and wrote the manuscript. JW, LS, and PM revised the manuscript. All authors have read and approved the final version of the manuscript.

## Funding

This study was part of a project that has received funding from the European Union's Horizon 2020 research and innovation program of Europe (No. 945698) and Zhejiang Province Science and Technology Department Key R&D Program of China (No. 2021C03097).

## Conflict of Interest

This study received funding from Venus Medtech. The funder was not involved in the study design, collection, analysis, interpretation of data, the writing of this article, or the decision to submit it for publication. The authors declare that the research was conducted in the absence of any commercial or financial relationships that could be construed as a potential conflict of interest.

## Publisher's Note

All claims expressed in this article are solely those of the authors and do not necessarily represent those of their affiliated organizations, or those of the publisher, the editors and the reviewers. Any product that may be evaluated in this article, or claim that may be made by its manufacturer, is not guaranteed or endorsed by the publisher.

## Abbreviations

AS: aortic stenosis
BAV: bicuspid aortic valve
CAD: computer aided design
CT: computed tomography
FEA: finite element analysis
HU: Hounsfield unit
DSCT: dual source computed tomography
TAV: tricuspid aortic valve
TAVR: transcatheter aortic valve replacement
TTE: transthoracic echocardiography.
